# Lessons learned: eight years at the helm of Biology Open

**DOI:** 10.1242/bio.037317

**Published:** 2018-08-15

**Authors:** Jordan W. Raff

**Affiliations:** Sir William Dunn School of Pathology, University of Oxford, South Parks Road, Oxford, OX1 3RE, UK


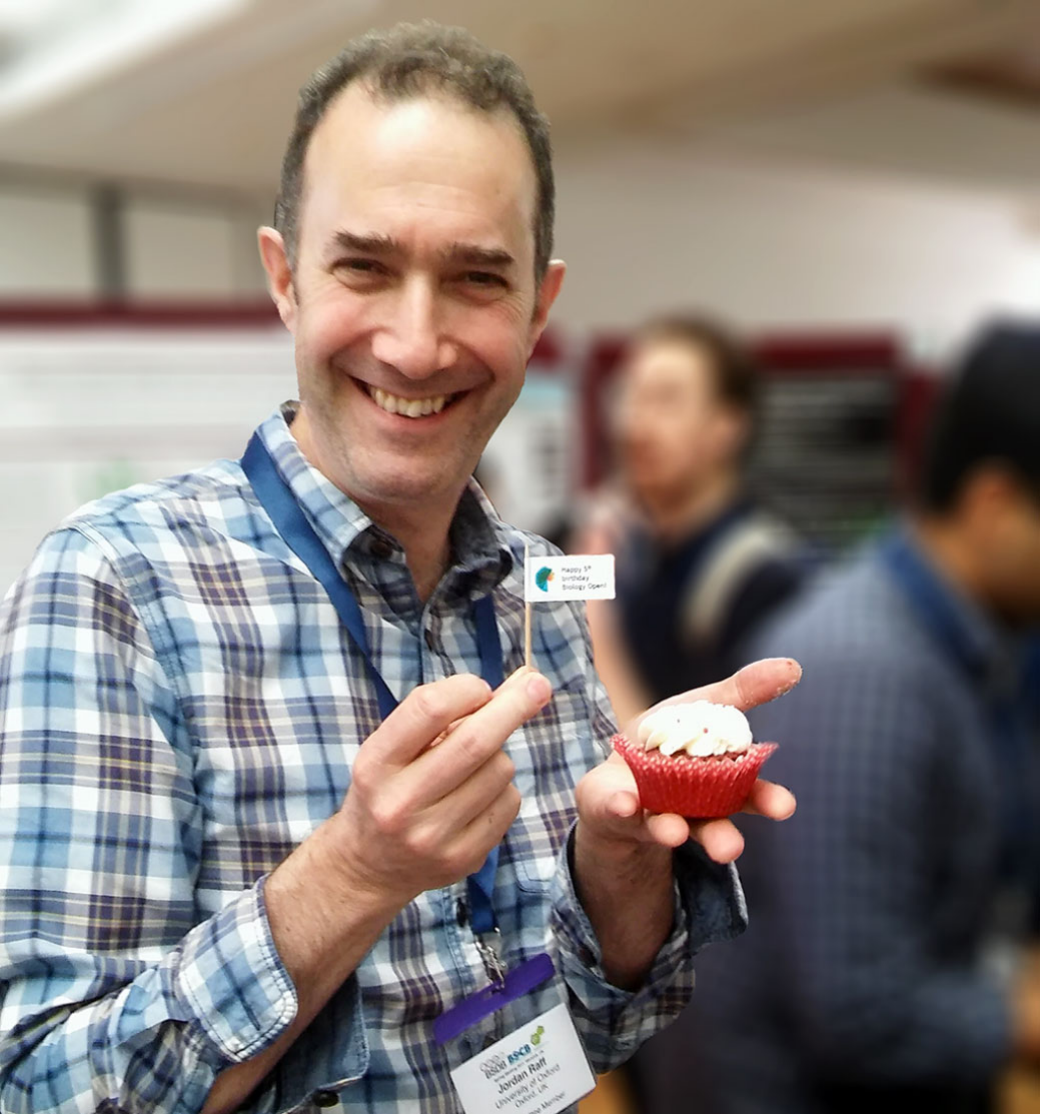


In 2011, I wrote an Editorial to introduce Biology Open (BiO) as a new journal from The Company of Biologists (Raff, 2012a). I quickly followed up with another Editorial explaining why I thought scientific publishing was going to change dramatically over the next few years. I also outlined some of the potential opportunities and dangers that this change might bring for the biomedical research community (Raff, 2012b). Now, after eight enjoyable years, I'm stepping down as Editor-in-Chief. It therefore seems a good time to reflect upon whether any of my initial musings have come to pass and what I have learned during this time.

My prediction that biomedical publishing would change rapidly appears to have been wide of the mark. When BiO was launched, many of the problems associated with publishing in the biomedical sciences were well known and widely discussed. Powerful people and institutions railed against the large profit margins of the major publishers of scientific papers, arguing that these profits were built on the backs of taxpayers who funded the science, paid for the scientists who review the papers, and then had to pay the publishers to read the papers. Many scientists also rebelled against the dominance of journal Impact Factors (IFs), which, although widely discredited, influenced so many aspects of a scientist's career, ensuring that only those who publish in high-impact journals are rewarded with jobs, grants and promotions. This rebellion led to the launch of the DORA declaration, of which The Company of Biologists was an early signatory (Raff, 2013). (This recently rejuvenated initiative recognises the need to improve the ways in which the outputs of scholarly research are evaluated.) Therefore, although there are signs that the publishing ecosystem is evolving, and the successful business models that will drive scientific publishing in the future remain uncertain, I would argue that these two major problems remain unresolved.

I also predicted that there was a real danger that the move from a subscription-based publishing system (whereby institutions pay a subscription to publishers for access to their journals) to an Open Access system (whereby authors pay publishers a fee to publish their work, which is then freely available to everyone) could have disastrous unintended consequences if the fundamental distortions of IFs were not dealt with first. Unfortunately, this prediction appears to have been more prescient. Our continuing obsession with IFs – even though this is often now expressly forbidden for tenure review and grant funding – has meant that publishers such as the Nature Publishing Group (NPG) and Cell Press/Elsevier have successfully launched a string of sister journals, many of which are Open Access and feed off the success of their high-profile parent journals. These new journals hoover up great swathes of the best papers that used to go to smaller, community-based, not-for-profit journals, creating a vicious feedback loop that simultaneously enhances the IFs of the new NPG/Elsevier journals and lowers the IFs of the journals of competing publishers that don't have such high-profile parents to drive the quality of their submissions. The electronic nature of these new journals means that there is no limit to the number of papers they can take from competing journals; the Open Access model ensures that the more papers they publish, the more money they make for their owners. Indeed, the seemingly inevitable high IF of these new journals means the owners can charge much higher fees than their rivals, as scientists are willing to pay more to publish in journals with a higher IF (it costs, for example, ∼$5000 to publish in Nature Communications or Cell Reports, compared to ∼$1500 to publish in PLoS One or BiO).

Thus, my fear that the rush to Open Access would end up consolidating the profits of the elite publishers that were lucky enough to have the most prestigious journals in their portfolio seems likely to have been justified, at least in the short-to-medium term. I fear there is a real danger that the meteoric rise of journals such as Nature Communications, Cell Reports and Scientific Reports will annihilate many of our most cherished community journals, some of which have been around for more than 100 years. Would this necessarily be a bad thing? I cannot be certain, but I think that a system whereby all credible journals are owned by a small handful of publishers who can dictate the price of publishing in these journals would be inherently risky; it is certainly not the model originally envisaged by early proponents of Open Access publishing.

Box 1. Recent developments and initiatives at Biology Open **First author interviews**

BiO is keen to support the next generation of biomedical scientists. The first authors of accepted articles now have the opportunity to talk about themselves and their research in more detail. In these popular ‘First person’ interviews, early-career researchers talk about their work in and out of the lab, the journeys that led them to where they are now and the issues they feel are priorities for early-career researchers.

**Continuous publication**

Issue 1 of 2018 was the first issue of BiO to be published under the continuous publication model. BiO has long posted peer-reviewed author manuscripts soon after acceptance. Now, the final version of the article is immediately published online, rather than waiting for the other articles in the issue to be completed, resulting in faster, free access to the article for our international community of readers. Our continuous publication issues reported an average of 33 days (median 31 days) between article acceptance and publication of the final version (compared with 38 days in 2017).

Readers should consider reviewing their BiO alert options, to ensure that they are receiving the content alerts they need, when they need them.

**Play-in-place movies**

BiO articles now feature play-in-place movies in the full-text online version of its articles. This makes videos an integral part of any article, making it easier and quicker for readers to access this type of content (see our play-in-place dolphin movies).

**Preprint-friendly policies and preLights**

BiO has had preprint-friendly policies since launch and, beginning in 2016, has had a two-way portal between BiO and bioRxiv so that authors submitting to BiO can simultaneously deposit their article in bioRxiv, and vice versa.

In February 2018, The Company of Biologists, the publisher of BiO, launched preLights, a new preprint highlighting service, run by the biological community.

preLights has a dedicated team of scientists from the community who regularly select, highlight and comment on preprints they feel are of interest to the biological community. You'll find a summary of each preprint, the reasons it was selected and the selector's thoughts on its significance. You might also see relevant comments from the preprints' authors and we encourage website visitors to join in the conversation.

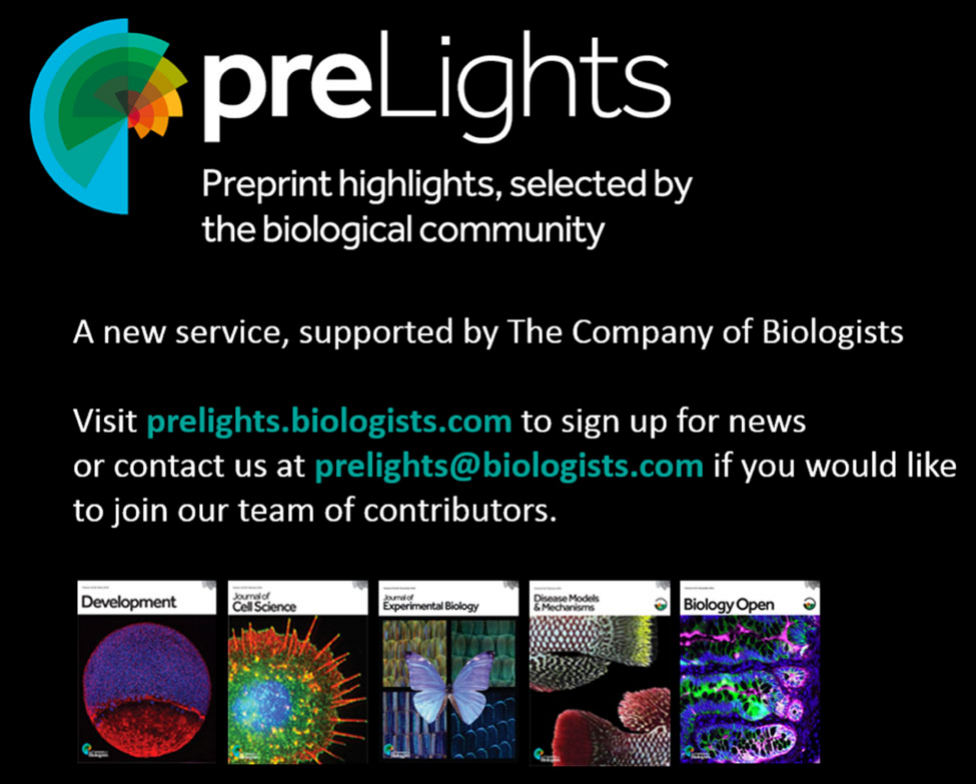

Is there anything we can do to prevent this outcome? The main problem remains our obsession with IFs. Until this problem is fixed, I see no realistic solution. The attraction of IFs is that they remain the easiest way to assess the impact of a scientist's work; we all know it is not a great way to assess the work's importance, but the alternative – to find the time to read a scientist's papers, evaluate the data and assess the importance of the conclusions – is, in the main, simply not going to happen. Much thought has gone into finding alternative metrics to measure the performance of researchers, but none have so far succeeded.

One strategy that could perhaps help is to encourage scientists to publicly comment on each other's work. In my field, there have been several papers published in high-profile journals over the years that I believe are flawed. I have never commented on them on the journals' websites, as it is generally not how we bioscientists do things. Would it have been better if I did? The authors might have valid answers to my concerns, which might help me, and perhaps other readers, understand their work better. If the authors agree that my concerns are important, this might spur them to further experiments; if they disagree, they can explain why. If such a system worked well, the important papers in a field might be recognised by common acclaim, no matter where they were published, whereas those papers that turned out to be more hype than substance might quickly be identified. If a paper weren't that important, it presumably wouldn't receive many comments. The rise of ‘Altmetrics’ (mainly a measure of how often a paper is mentioned on social and mainstream media) is not nearly nuanced enough to provide this type of analysis, but the rise of preprint servers such as bioRxiv might provide a forum in which providing feedback becomes more acceptable.

Such a system clearly has several potential pitfalls, and it would take time to evolve into a useful way to quickly and accurately judge the value of a paper's contribution. Nevertheless, the next generation of scientists are much more comfortable in an online world, where commenting on other people's contributions is the norm. Perhaps we scientists should all be encouraging this habit?

In my first Editorial, I also expressed the hope that BiO would be something special. I thought that, by having a not-for-profit publisher such as The Company of Biologists and a team of academic Editors close to their publishing communities, BiO could establish a meaningful connection with our authors and readers. Although I was perhaps naïve in this aspiration, I like to think that it was not entirely a pipedream. Through handling submissions to BiO, I have interacted with many hundreds of authors and reviewers. I have always tried to give helpful advice, even when rejecting a paper without sending it for review [such as when BiO was among the Open Access journals ‘tested’ by a spoof article devised by a journalist (Hackett et al., 2013)]. These interactions have almost always been good natured and respectful and, although it has been hard work, I have benefitted greatly from the experience. I hope that many of our authors will feel the same way, although the dramatic (and continuing) increase in submissions to BiO made this challenging at times.

Finally, I want to express my extreme gratitude to all those who have helped BiO. These include the authors who have submitted papers to the journal, the hard-working team of academic Editors who have handled them and all the reviewers who have reviewed them and the excellent editorial team at The Company of Biologists who have made my job so enjoyable over the years.

I wish my successor, Steven Kelly, the best of luck. From his accompanying Editorial (Kelly, 2018), I hope you can see why I am so confident that his enthusiasm and tactical nous will push the journal to new heights in this very competitive publishing environment.
